# Supplementary material for: Sialic Acid Identity Modulates Host Tropism of Sialoglycan-binding Viridans Group Streptococci
Source: bioRxiv. 2025 Jun 26:2025.06.24.660003. Preprint. [Version 1] doi: 10.1101/2025.06.24.660003 (PMC12262549; doi:10.1101/2025.06.24.660003)
Supplement: Supplement 1 [file media-1.pdf]

Sialic Acid Identity Modulates Host Tropism of Sialoglycan-binding Viridans Group Streptococci

KeAndreya M. Morrison[1], Rupesh Agarwal[2,¶], Haley E. Stubbs[3], Hai Yu[4] , Stefan Ruhl[5], Xi Chen[4], Paul M Sullam[6], Barbara A Bensing[6], Jeremy C. Smith[2,7], T. M. Iverson[1,8,*]

[1]Department of Pharmacology, School of Graduate Studies, Meharry Medical College, Nashville, TN, USA
[2]UT/ORNL Center for Molecular Biophysics, Oak Ridge National Laboratory, TN, USA
[3]Chemical and Physical Biology Program, Vanderbilt University, Nashville, TN, USA
[4]Department of Chemistry, University of California, Davis, CA, USA
[5]Department of Oral Biology, University at Buffalo, Buffalo, NY, USA
[6]Division of Infectious Diseases, Veterans Affairs Medical Center, Department of Medicine, University of California, San Francisco, and the Northern California Institute for Research and Education, San Francisco, CA, USA
[7]Department of Biochemistry and Cellular and Molecular Biology, University of Tennessee, Knoxville, TN
[8]Departments of Pharmacology and Biochemistry, Vanderbilt University, Nashville, TN, USA

¶Current address:
*Correspondence to: tina.iverson@vanderbilt.edu

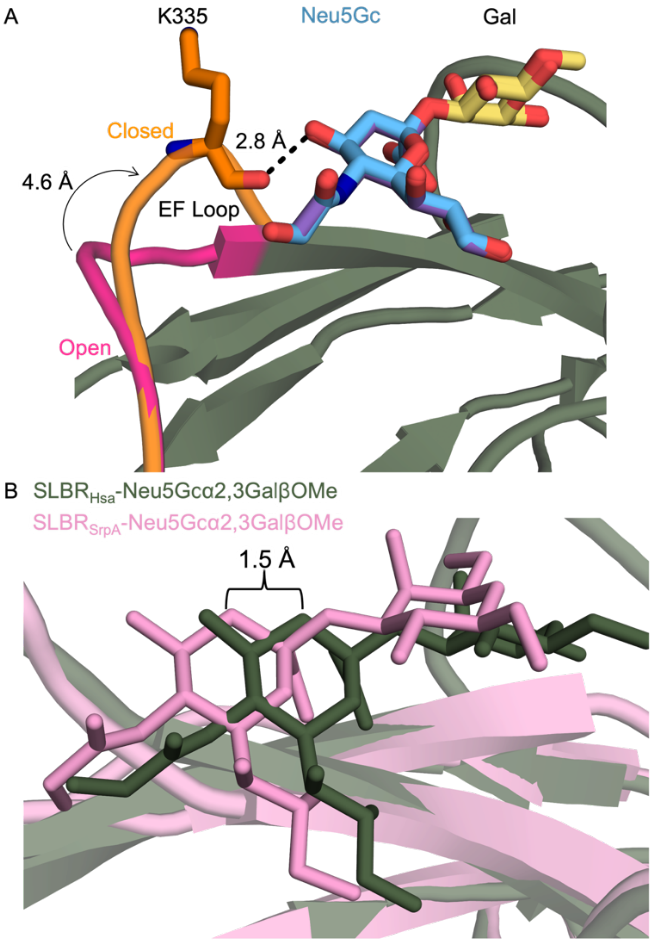

Supplementary Figure 1 Aspects of ligand binding in SLBR$_{Hsa}$ and SLBR$_{SrpA}$. A) In SLBR$_{Hsa}$-Neu5Gc, the EF loop (*orange*) adopts a closed conformation that allows for a hydrogen bond between the SLBR$_{Hsa}^{K335}$ carbonyl and O4 of Neu5Gc. In SLBR$_{Hsa}$-Neu5Ac, the EF loop (*hot pink*) remains in the open position. **B)** SLBR$_{SrpA}$[1] and SLBR$_{Hsa}$ each bind glycan above the F strand, however there is a lateral shift in position of 1.5 Å with respect to the ΦTRX motif. Shown is each SLBR–Neu5Gc costructure overlaid based on Cα atom positions.

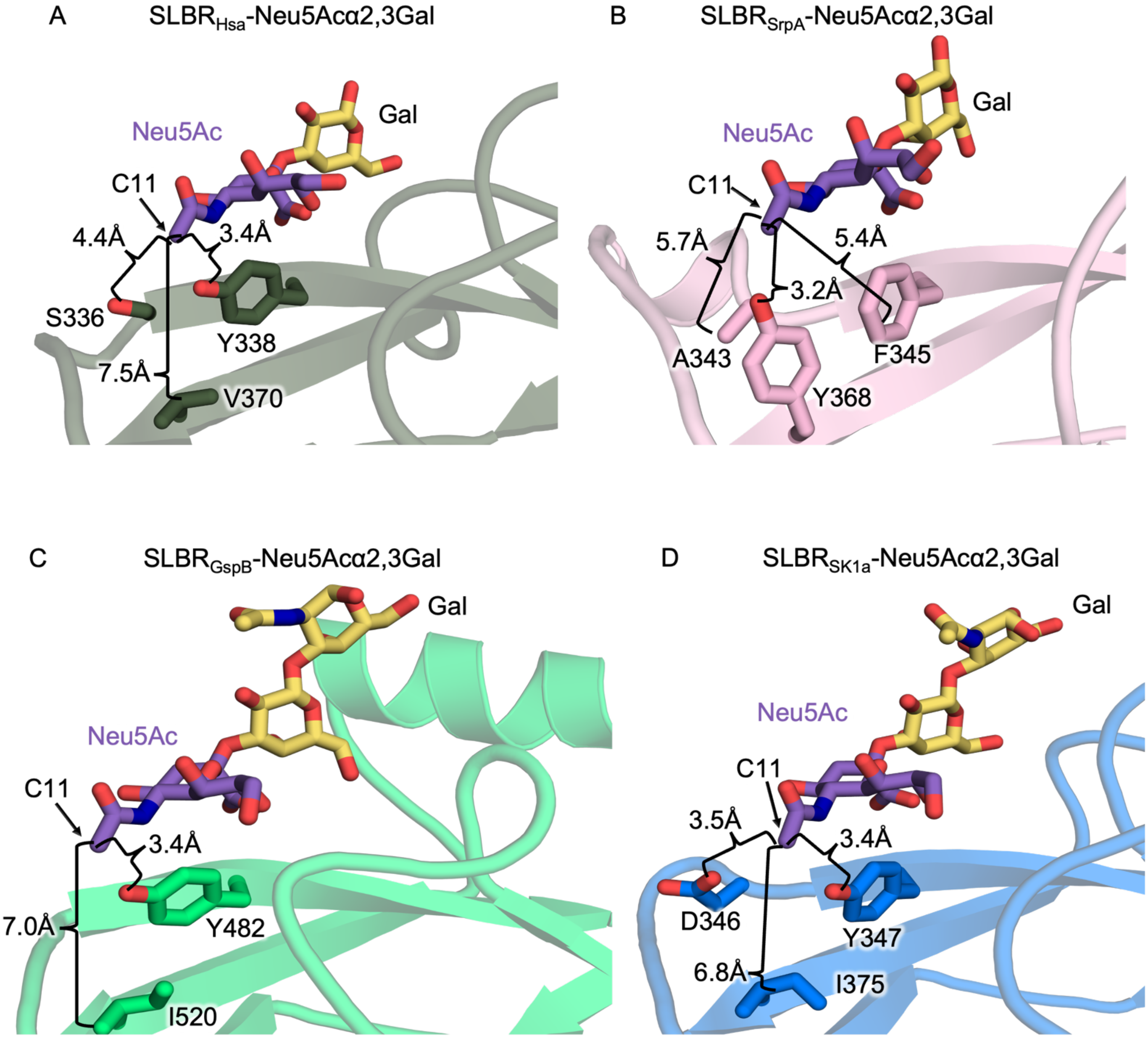

Supplementary Figure 2 Comparison of Neu5Ac-bound SLBRs. A) $SLBR_{Hsa}$, **B)** $SLBR_{SrpA}$[1], **C)** $SLBR_{GspB}$[2], and **D)** $SLBR_{SK1a}$[3]. Distances between adjacent non-bonding atoms are shown with brackets.

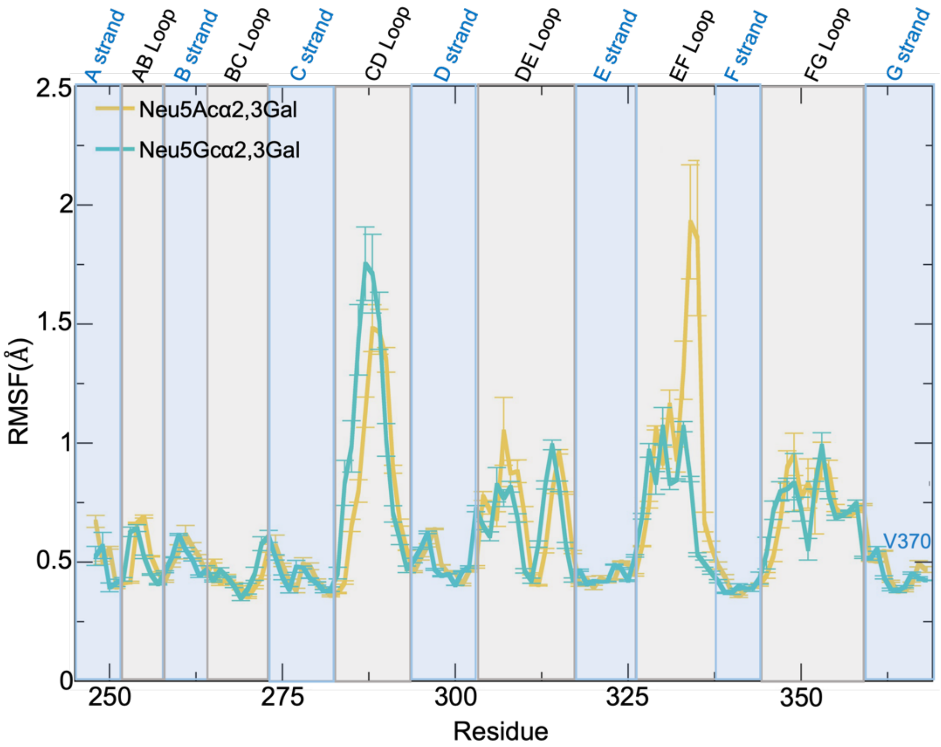

Supplementary Figure 3 RMSF of the SLBR$_{Hsa}$ bound to Neu5Acα2-3Gal or Neu5Gcα2-3Gal. RMSF plot for SLBR$_{Hsa}$ residues during MD simulations show backbone flexibility in the presence of Neu5Ac (gold) or Neu5Gc (*cyan*). Sequence regions corresponding to the CD-, EF-, and FG-loops are highlighted with *grey boxes*, while the secondary structural elements are highlighted with *blue boxes*.

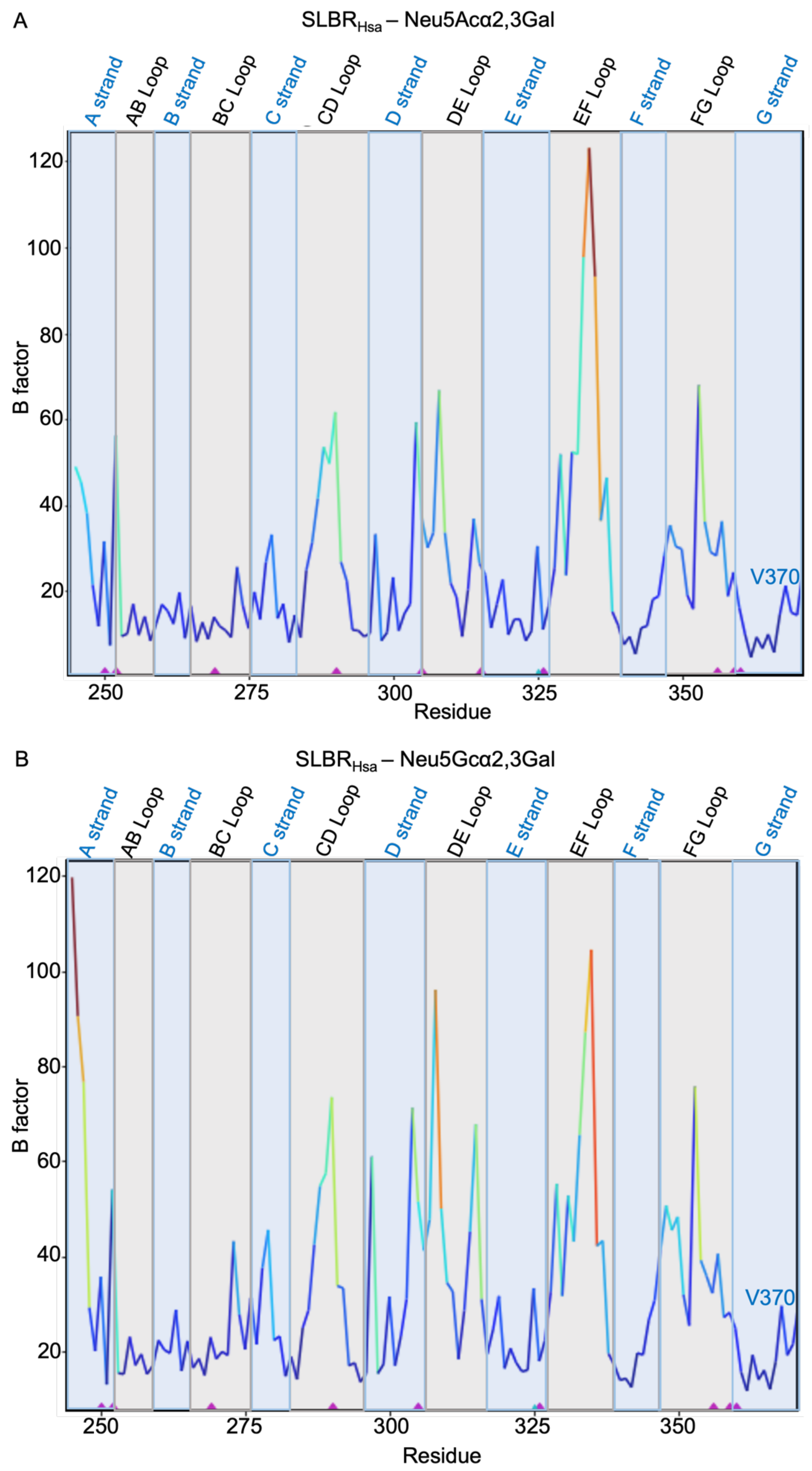

Supplementary Figure 4 Crystallographic temperature factors of SLBR$_{Hsa}$. A) Neu5Ac and **B)** Neu5Gc. The temperature factors of the CD-, EF-, and FG-loops are highlighted with *grey boxes*. The temperature factors of

the secondary structural elements are highlighted with *blue boxes*. Residues are colored by B-factor magnitude, with warmer colors representing a higher temperature factor.

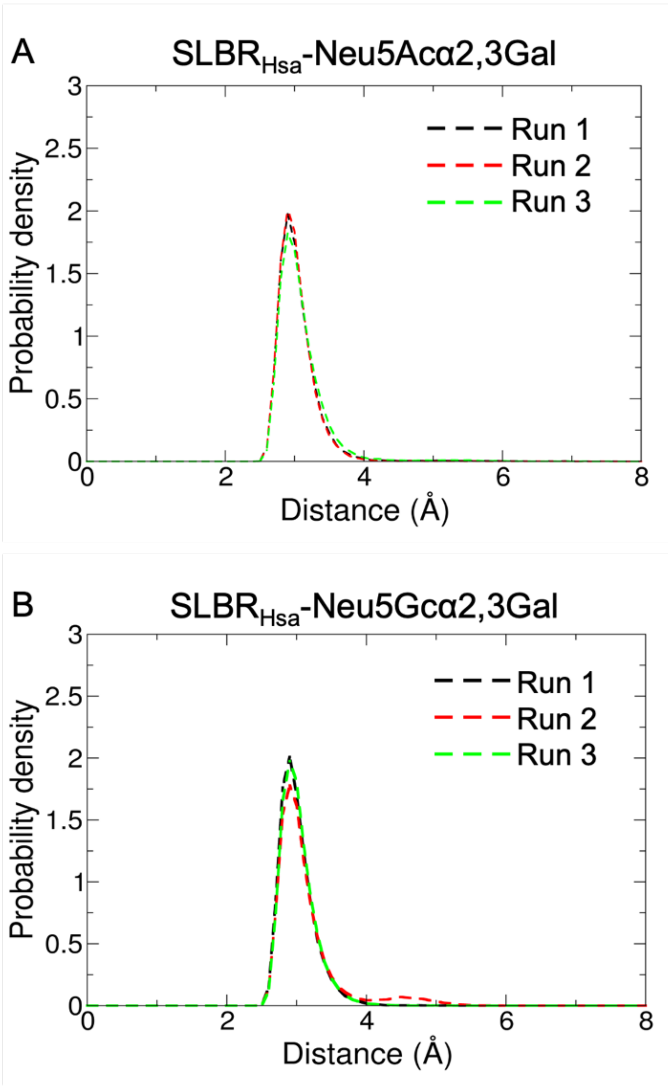

Supplementary Figure 5 Probability distributions of the distance of the EF-loop in SLBR$_{Hsa}$. Distance between SLBR$_{Hsa}^{K335}$ backbone carbonyl and Neu5Gc-O4 in simulations of SLBR$_{Hsa}$ bound to disaccharides terminated in **A)** Neu5Ac and **B)** Neu5Gc. The ~3 Å distance is consistent with a hydrogen-bonding interaction that accompanies EF loop closure of the ligand for the duration of the simulation. All simulations were performed in triplicate.

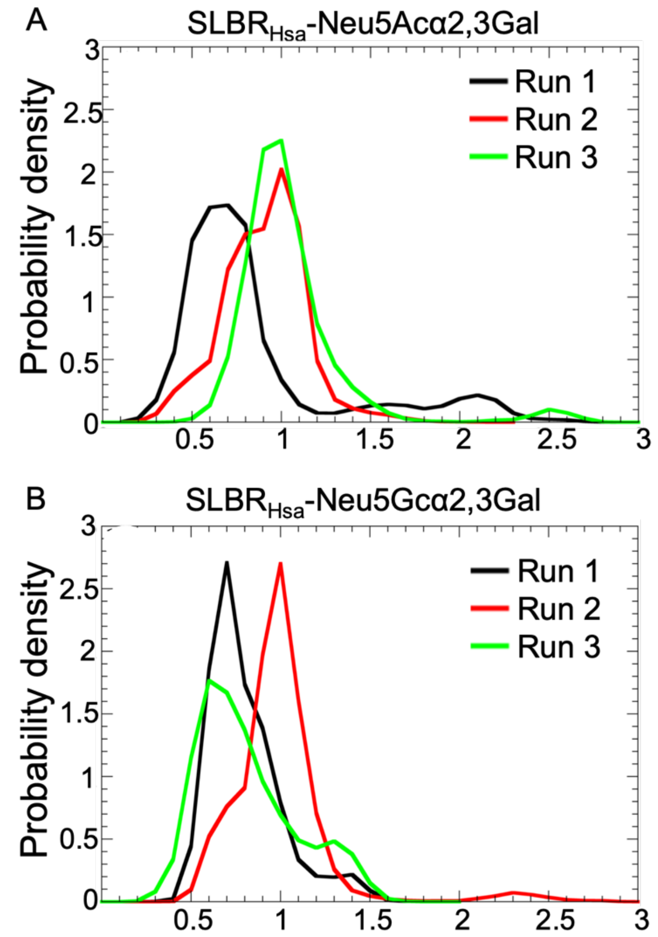

Supplementary Figure 6 Probability distribution RMSD of disaccharides bound to $SLBR_{Hsa}$. A) Neu5Ac and **B)** Neu5Gc. Three independent molecular dynamics simulations were performed. Mean RMSD Neu5Ac = 0.85 Å (±0.14 Å). Mean RMSD Neu5Gc = 0.83 Å (±0.15 Å).

Supplementary Information References

1 Bensing, B. A. *et al.* Structural Basis for Sialoglycan Binding by the Streptococcus sanguinis SrpA Adhesin. *J Biol Chem* **291**, 7230-7240, doi:10.1074/jbc.M115.701425 (2016).

2 Pyburn, T. M. *et al.* A structural model for binding of the serine-rich repeat adhesin GspB to host carbohydrate receptors. *PLoS Pathog* **7**, e1002112, doi:10.1371/journal.ppat.1002112 (2011).

3 Stubbs, H. E. *et al.* Tandem sialoglycan-binding modules in a Streptococcus sanguinis serine-rich repeat adhesin create target dependent avidity effects. *J Biol Chem* **295**, 14737-14749, doi:10.1074/jbc.RA120.014177 (2020).
